# Apoptosis is a generator of Wnt-dependent regeneration and homeostatic cell renewal in the ascidian *Ciona*

**DOI:** 10.1242/bio.058526

**Published:** 2021-04-23

**Authors:** William R. Jeffery, Špela Gorički

**Affiliations:** 1 Department of Biology, University of Maryland, College Park, MD 20742, USA; 2 Station Biologique, Roscoff 29680, France; 3 Scriptorium Biologorum LLC, Murska Sobota 9000, Slovenia

**Keywords:** Regeneration, Homeostasis, *Ciona*, Apoptosis, Wnt signaling, Stem cells

## Abstract

In the ascidian *Ciona intestinalis*, basal body parts regenerate distal structures but distal body parts do not replace basal structures. Regeneration involves the activity of adult stem cells in the branchial sac, which proliferate and produce migratory progenitor cells for tissue and organ replacement. Branchial sac-derived stem cells also replenish recycling cells lining the pharyngeal fissures during homeostatic growth. Apoptosis at injury sites occurs early during regeneration and continuously in the pharyngeal fissures during homeostatic growth. Caspase 1 inhibitor, caspase 3 inhibitor, or pan-caspase inhibitor Z-VAD-FMK treatment blocked apoptosis, prevented regeneration, and suppressed branchial sac growth and function. A pharmacological screen and siRNA-mediated gene knockdown indicated that regeneration requires canonical Wnt signaling. Wnt3a protein rescued both caspase-blocked regeneration and branchial sac growth. Inhibition of apoptosis did not affect branchial sac stem cell proliferation but prevented the survival of progenitor cells. After bisection across the mid-body, apoptosis occurred only in the regenerating basal fragments, although both fragments contained a part of the branchial sac, suggesting that apoptosis is unilateral at the wound site and the presence of branchial sac stem cells is insufficient for regeneration. The results suggest that apoptosis-dependent Wnt signaling mediates regeneration and homeostatic growth in *Ciona*.

## Abstract (precis)

**Summary:** Apoptosis induces Wnt-dependent regeneration and homeostatic cell renewal in *Ciona*. Apoptosis is required for stem cell survival and is absent in non-regenerating body parts, suggesting a role in asymmetrical regeneration.

## INTRODUCTION

Ascidians are well known for their inability to replace ablated embryonic cells and thus are often described as exemplary models for determinate or mosaic development (Jeffery, 2001; Nishida, 2002). After metamorphosis, however, ascidian juveniles and adults show impressive capacities for regeneration of injured body parts. Colonial ascidians, such as *Botryllus*, can reform the entire zooid body, including all somatic and germ line cells, from small parts of the basal vasculature or even single vascular cells, a phenomenon known as whole body regeneration (Freeman, 1964; Tiozzo et al., 2008; Voskoboynik et al., 2008). After evisceration, the solitary ascidian *Polycarpa* can replace the entire digestive system and reform a functional branchial sac (Shenkar and Gordon, 2015). Most solitary ascidians can also regenerate the oral and atrial siphons after amputation (Auger et al., 2010; Jeffery, 2015a; Gordon et al., 2019). And uniquely among the chordates, many ascidian species can regenerate the central nervous system, including the neural complex and associated endocrine-like organs (Schultze, 1899; Dahlberg et al., 2009; Medina et al., 2014; Gordon and Shenkar, 2018). As solitary ascidians age, the capacity for regeneration gradually fades and eventually disappears (Dahlberg et al., 2009; Auger et al., 2010; Jeffery, 2012; Gordon and Shenkar, 2018). Although the regeneration capacities of ascidians have been well described, the molecular and cellular mechanisms of regeneration are still incompletely understood (Kassmer et al., 2019).

*Ciona* (*Ciona intestinalis* and *Ciona* *robusta*) is the most frequently studied solitary ascidian model for regeneration studies (Schultze, 1899; Hirschler, 1914; Jeffery, 2015a). The favorability of this model for regeneration research is due to its relatively large size, body transparency (during youth), and resilience to many different surgical operations. *Ciona* exhibits asymmetric body regeneration, a process in which severed basal body fragments can regenerate missing distal parts, such as the siphons and neural complex, but severed distal fragments are unable to regenerate basal parts, such as the heart, gonads, and other visceral organs, and eventually perish (Hirschler, 1914; Jeffery, 2015b). This type of regeneration contrasts to that of planarians (Reddien and Sanchez-Alvarado, 2004) and *Hydra* (Reddy et al., 2019), in which complete severance of the body leads to the regeneration of both fragments. A requirement for ascidian regeneration seems to be the inclusion of the branchial sac, a massive pharyngeal organ, in a regenerating body fragment (Hirschler, 1914; Jeffery, 2015b). The branchial sac is punctuated by perforations called pharyngeal fissures, which are lined with ciliated cells that propel water through the body cavities for filter feeding and gas exchange (Manni et al., 2002). *Ciona* increase in size rapidly during adult life, largely due to the substantial growth of the branchial sac, which involves the elongation, splitting, and multiplication of the pharyngeal fissures and adjacent tissues (Millar, 1953). Beginning with only two pharyngeal fissures in recently metamorphosed juveniles, mature adult *Ciona* eventually form a branchial sac consisting of hundreds of pharyngeal fissures aligned in multiple rows perpendicular to the longitudinal axis.

The adult branchial sac also contains multiple transverse vessels with lymph nodes containing niches of adult stem cells (Jeffery, 2015b). The stem cells of the branchial sac divide to produce migratory progenitor cells responsible for replacing distal body parts, including the siphons and neural complex, the repair of wounds, and the replacement of ciliated cells in the pharyngeal fissures, which turnover rapidly during branchial sac growth (Jeffery, 2015b, 2019). A short exposure of regenerating *Ciona* to the cell proliferation marker EdU strongly labels the branchial sac stem cells, and the resulting progenitor cells can be chased into the sites of wounds and regenerating organs (Jeffery, 2015b, 2019). The progenitor and stem cell migrations specifically target the sites of injury or cell replacement, and in cases of multiple injured sites, each of them, but not the uninjured sites, are invaded by cells derived from the branchial sac stem cell niches. During homeostatic growth the same stem cell niches also produce progenitor cells for replenishment of the recycling ciliated cells in the pharyngeal fissures (Jeffery, 2019). Oral siphon regeneration in *Ciona* involves the upregulation of a suite of genes at the site of amputation, including those coding for microRNAs and members of the Notch pathway, such as Delta, Notch, and Fringe, Wnt signaling system components, apoptosis regulators, cell guidance factors such as netrins, and proteins involved in tissue repair (Hamada et al., 2015; Gorički, unpublished; Spina et al., 2017). However, the system that activates cell proliferation in the stem cell niches and directs the migration of progenitor cells to their distal body and pharyngeal fissure targets has not been identified.

The diverse sites in the *Ciona* body targeted by branchial sac stem cells have one commonality: they are all regions of extensive and transient apoptotic cell death (Jeffery, 2019). Apoptosis begins at the margin of the excision early after siphon amputation, extirpation of the neural complex, or wounding, and appears to occur continuously during the rapid turnover and replacement of cells lining the pharyngeal fissures during homeostatic growth. Apoptosis is also linked with regeneration in other animals (Bergmann and Steller, 2010), including the regenerating head in *Hydra* (Chera et al., 2009) and the tail in *Xenopus* tadpoles (Tseng et al., 2007), and appears to induce a signaling cascade leading to cell proliferation (Fogarty and Bergmann, 2017). In *Hydra*, a Wnt ligand is produced by dying cells at the wound site, and apoptosis-driven Wnt signaling has been shown to be an integral part of the head regeneration process (Chera et al., 2009). The Wnt signaling pathway has also been shown to be involved in tissue and organ regeneration in other animals (Whyte et al., 2012).

In this investigation, we have employed caspase inhibitors to determine the roles of apoptosis, Wnt signaling, and progenitor cell targeting during *Ciona* asymmetric regeneration and branchial sac homeostasis. We show that apoptosis is required for distal regeneration and normal homeostatic cell replacement in the branchial sac, Wnt signaling is involved in both processes, exogenously applied Wnt ligand rescues normal regenerative and homeostatic activities if apoptosis is inhibited, and apoptosis at the wound site is required for the survival, rather than the proliferation, of progenitor cells in the branchial sac stem cell niches.

## RESULTS

### Apoptosis is required for oral siphon regeneration

**Fig. 1.**
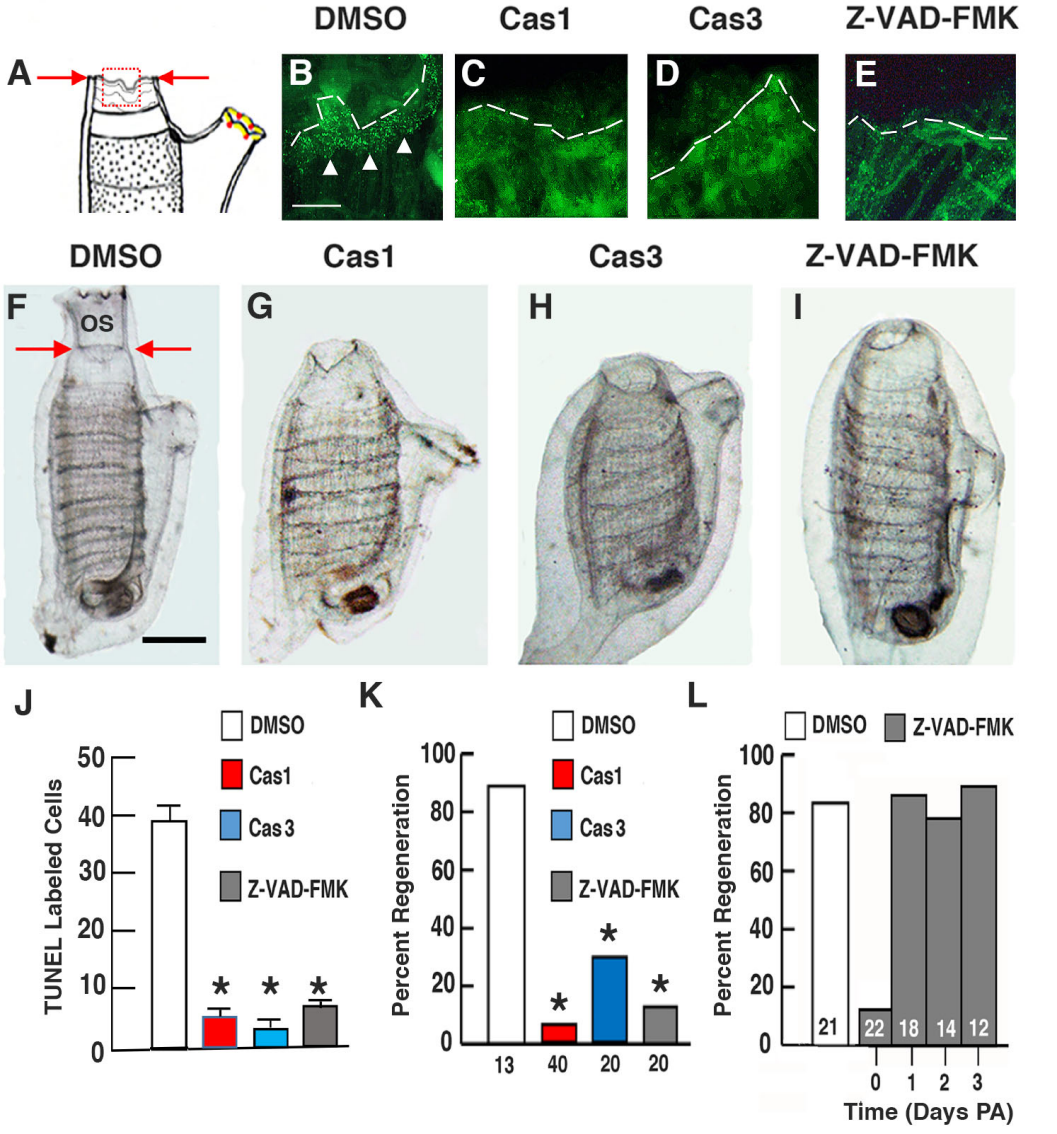
**Apoptosis is required for distal body regeneration.** (A) An illustration of a regenerating animal at 1-day post-amputation showing the region of the siphon margin (red square) in which TUNEL labeling was determined in B–E. Arrows show the position of amputation. (B–E) Animals with amputated oral siphons assayed by TUNEL labeling (arrowheads) at the amputation margin (dashed lines) after treatment with (B) DMSO (control), (C) caspase 1 inhibitor, (D) caspase 3 inhibitor, or (E) pan-caspase inhibitor Z-VAD-FMK at 12 h PA. Scale bar: 20 µm; magnification is the same in B–E. (F–I) Oral siphon regeneration assayed at 6 days PA after continuous treatment since amputation with (F) DMSO (control), (G) caspase 1 inhibitor, (H) caspase 3 inhibitor, or (I) pan-caspase inhibitor Z-VAD-FMK. Arrows in F show position of amputation. OS, oral siphon. Scale bar: 100 µm; magnification is the same in F–I. (J) Bar graphs comparing TUNEL labeling along the anterior margins of amputated siphon stumps in DMSO, caspase 1 inhibitor, caspase 3 inhibitor, and pan-caspase inhibitor Z-VAD-FMK treated animals with amputated oral siphons at 12 h PA. *N*=6 for each bar. Error bars: s.e.m. Asterisks indicate significant differences at *P*<0.001 between the control and caspase inhibitor treated animals. Statistics by one-way ANOVA and post-hoc Tukey with Bonferroni correction. (K) Bar graphs showing the percentage of regeneration at 6 days PA after continuous treatment with DMSO (control), caspase 1 inhibitor, caspase 3 inhibitor, or pan-caspase inhibitor Z-VAD-FMK since the time of amputation. Numbers of animals are indicated at the bases of the bars. Asterisks indicate significant differences at *P*<0.001 between the control and caspase inhibitor treated animals. Statistics by χ^2^ test and post-hoc Fisher's exact test with Bonferroni correction. (L) Bar graphs showing the relationship between the beginning of pan-caspase inhibitor Z-VAD-FMK treatment after oral siphon amputation and the percentage of regeneration at 6 days PA. Numbers of animals are indicated within the bars. Asterisk indicates significant difference at *P*<0.001 between the control and day 0 caspase inhibitor treated animals. Statistics by χ^2^ test and post-hoc Fisher's exact test with Bonferroni correction. Each experiment was replicated at least three times.

To determine whether apoptosis is required for regeneration, oral siphons were amputated, and the amputees were immediately treated with caspase 1 inhibitor, caspase 3 inhibitor, pan-caspase inhibitor Z-VAD-FMK, or dimethylsulfoxide (DMSO), which was used as a control (Fig. 1A–E,J). At 12 h post-amputation (PA), some of the amputated animals were fixed and subjected to Terminal deoxynucleotidyl transferase dUTP nick end labeling (TUNEL) to determine the effects on apoptosis at the wound sites. At this early stage in the regeneration process (Auger et al., 2010), the wound epidermis had not formed and the contracted oral siphon stumps still showed irregular margins (Fig. 1A–E). In the controls, a band of TUNEL labeling was detected at the amputated margin of the oral siphon (Fig. 1B), as described previously (Jeffery, 2019), but TUNEL labeling was significantly reduced in the amputees treated with caspase inhibitors (Fig. 1B–E,J), showing that apoptosis was suppressed. At 6 days PA, the inhibitor treated and control amputees were assayed for regeneration using re-growth of the oral siphon, differentiation of new circular muscle bands (CMB) (Jeffery, 2015b), and re-appearance of oral siphon pigment organs (OPO) (Auger et al., 2010) as criteria. Re-growth of the oral siphon, differentiation of multiple rows of new CMBs, and formation of the conventional number of 8 OPO, were seen in most of the controls (Fig. 1F,K), whereas most of the caspase-inhibitor treated amputees lacked these markers (Fig. 1G–I,K). Apoptosis is a transient step in oral siphon regeneration, which begins shortly after amputation and persists for about 1-day PA (Jeffery, 2019). The pan-caspase inhibitor Z-VAD-FMK was effective in suppressing regeneration when treatment was initiated immediately following oral siphon amputation but not after 1–3 days PA (Fig. 1L), thus linking caspase suppression of regeneration to the period of apoptosis and showing that caspase inhibitors have no effects on regeneration outside of the apoptosis period. These results indicate that apoptosis is required for oral siphon regeneration in *Ciona*.

### Apoptosis is required for branchial sac homeostasis and function

**Fig. 2.**
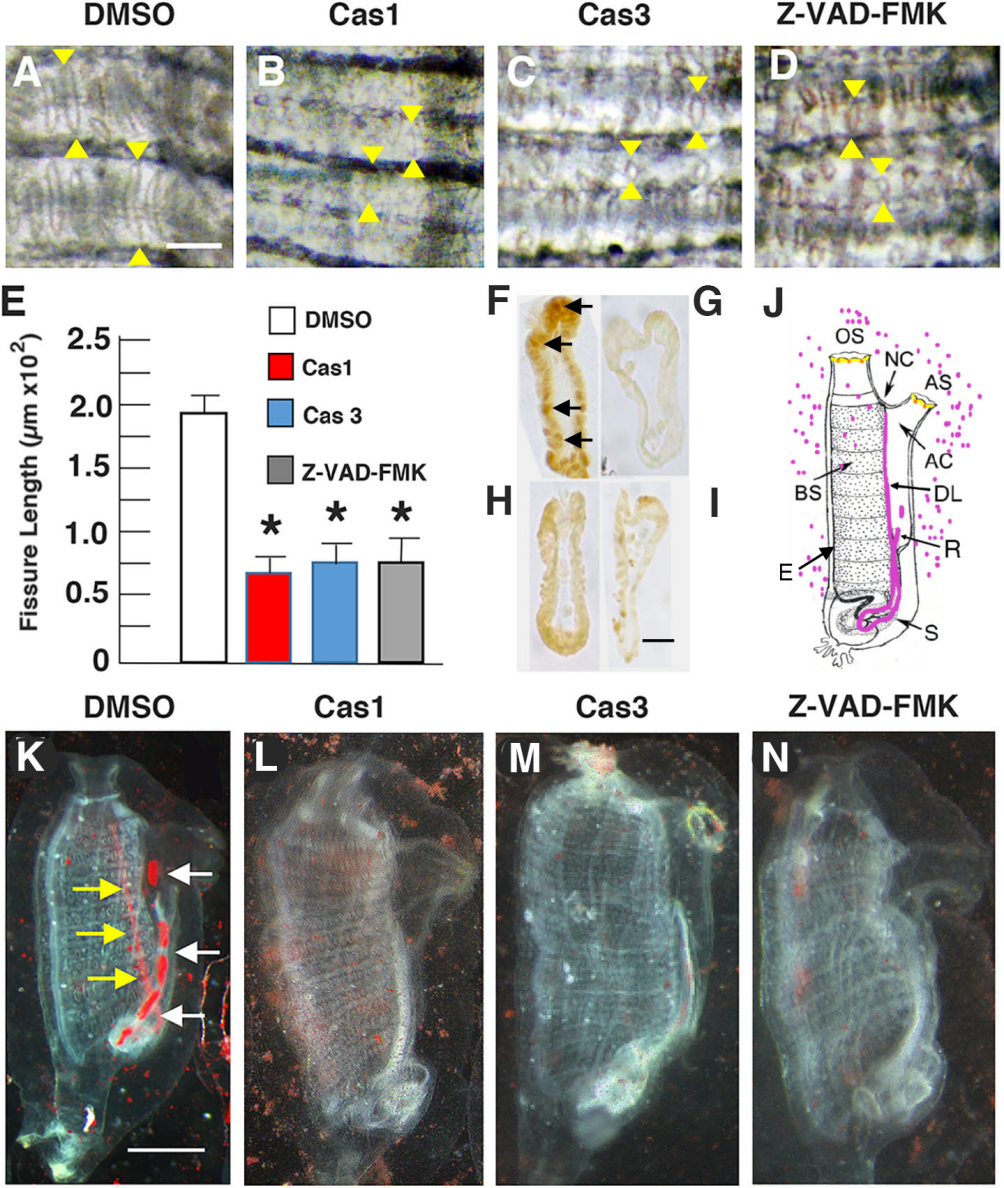
**Apoptosis is required for branchial sac homeostasis and function.** (A–D) Branchial sac fissures after 6-day treatment with (A) DMSO (control) (B) caspase 1 inhibitor, (C), caspase 3 inhibitor, or (D) pan-caspase inhibitor Z-VAD-FMK. Arrowheads: distal (top) and proximal (bottom) ends of the fissures. Scale bar: 15 µm; magnification is the same in A–D. (E) Bar graphs showing the mean lengths of branchial sac fissures in caspase inhibitor and DMSO (control) treated animals. *N*=12 for each bar. Error bars: s.e.m. Asterisks indicate significant difference at *P*=0.004 between the control and caspase inhibitor treated animals. Statistics by one-way ANOVA and post-hoc Tukey with Bonferroni correction. (F–I) Sections of TUNEL assayed pharyngeal fissures of (F) DMSO (control), (G) caspase 1 inhibitor-, (H) caspase 3 inhibitor, or (I) pan-caspase inhibitor Z-VAD-FMK treated animals. Arrows in F: TUNEL labeled cells. Scale bar: 5 µM; magnification is the same in all frames. (J) Diagram illustrating the carmine particle assay for branchial fissure function. Carmine particles shown by magenta dots and colored organs in the body. OS, oral siphon; AS, atrial siphon; NC, neural complex; BS, branchial sac; DL, dorsal lamina; S, stomach; R, rectum; AC, atrial cavity. (K–N) Carmine particle assay of animals treated with (K) DMSO, (L) caspase 1 inhibitor, (M) caspase 3 inhibitor, or (N) pan-caspase inhibitor Z-VAD-FMK. Scale bar: 110 µm; magnification is the same in K–N. Arrows in K show carmine particles concentrated in the dorsal lamina (yellow arrows) and rectum (white arrows). Each experiment was replicated at least three times.

Apoptosis also occurs during the homeostatic replacement of ciliated cells lining the pharyngeal fissures of the branchial sac (Jeffery, 2019). To determine if apoptosis is required for branchial sac homeostasis, the length of pharyngeal fissures was compared in animals treated with caspase 1, caspase 3, pan-caspase Z-VAD-FMK inhibitors, or DMSO for 6 days (Fig. 2A–E). TUNEL labeling showed that apoptotic cells were abundant in the pharyngeal fissures of the controls (Fig. 2F), but reduced or eliminated in animals treated with the caspase inhibitors (Fig. 2G–I). The pharyngeal fissures of the controls were organized in rows and elongated columns typical of growing *Ciona* (Millar, 1953; Manni et al., 2002) (Fig. 2A,E), but the pharyngeal fissures of animals treated with caspase inhibitors were smaller and organized haphazardly (Fig. 2B–E), suggesting that inhibition of apoptosis affected pharyngeal fissure growth and organization.

To determine the effect of caspase inhibitors on branchial sac function, a filtration assay was developed using carmine particles (Fig. 2J). In this assay, animals were treated with the caspase inhibitors, then incubated in Millipore Filtered Sea Water (MFSW) containing suspended carmine particles, and several hours later the accumulation of carmine particles was examined in the body. During ascidian filter feeding, food particles are trapped in a secretion from the endostyle, which is located on the ventral side of the branchial sac, and become concentrated in the dorsal lamina, which is located on the opposite side of the branchial sac, where a food bolus is formed and passed into the stomach and intestinal tract (Pennachetti, 1984; Petersen and Svane, 2002). The fecal pellets collect in the rectal tube and are ultimately expelled through the atrial siphon (Fig. 2J). The controls showed carmine particles concentrated in the dorsal lamina and rectum (Fig. 2K), indicative of active branchial sac filtration, whereas carmine particles did not accumulate in the body of animals treated with the caspase inhibitors (Fig. 2L–N), suggesting malfunction of the filtration process. The results suggest that apoptosis in the pharyngeal fissure cells is required for normal growth and function of the branchial sac.

### Role of Wnt signaling in oral siphon regeneration

**Fig. 3.**
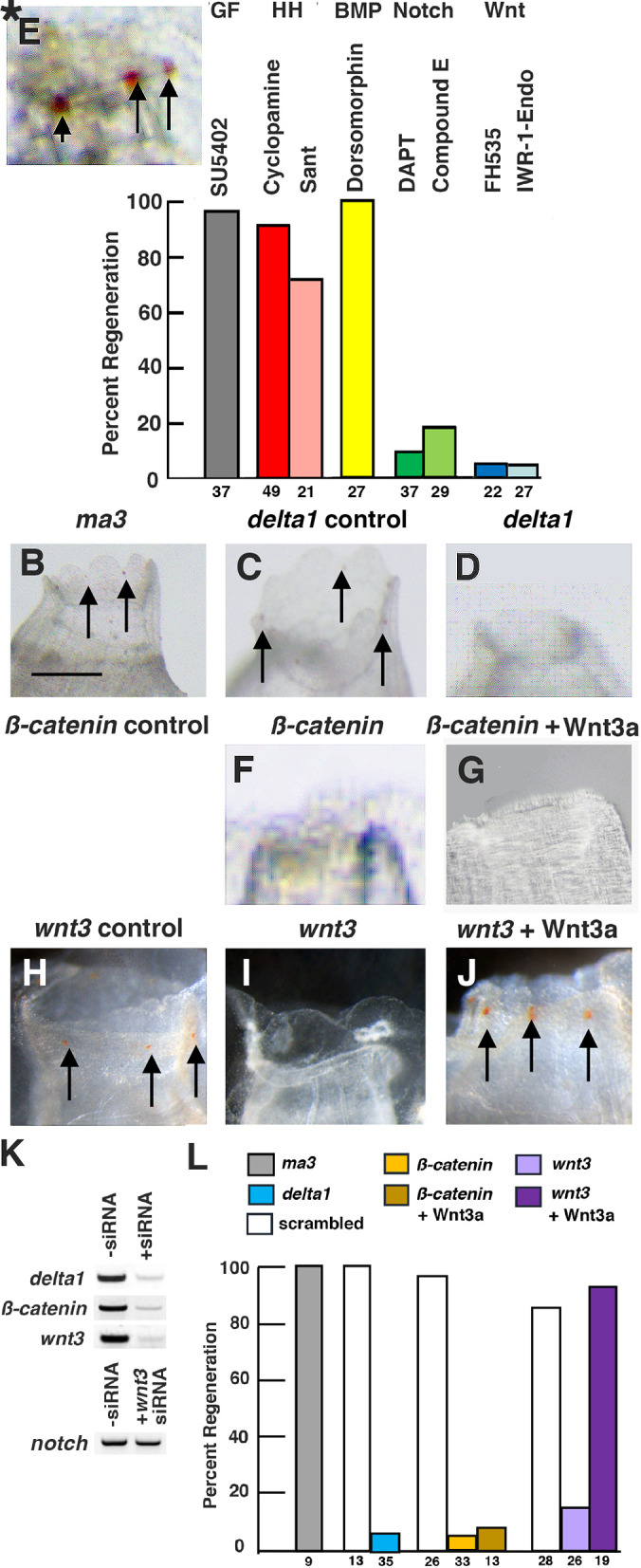
**Pharmacological screen and siRNA effects on oral siphon regeneration.** (A) Bar graphs showing the effects of signaling system inhibitors on percent oral siphon regeneration. Percent oral siphon regeneration was determined with respect to controls treated with DMSO. The inhibitors and affected signaling systems are shown at the top of the bars. The numbers of animals assayed are shown at the bottom of the bars. Black asterisks indicate significant differences at *P*<0.001 between the control and inhibitor treated animals. Red asterisk indicates significant difference at *P*<0.01. Statistics by χ^2^ test and post-hoc Fisher's exact test with Bonferroni correction. (B–J) Effects of siRNA on oral siphon regeneration. (B,C,E,H) Normal regeneration after treatment with (B) *ma3* siRNA, (C) scrambled *delta1* siRNA, (E) scrambled *ß-catenin* siRNA, or (H) scrambled *wnt3* siRNA. (D,F,G,I) Suppression of regeneration after treatment with (D) *delta1* siRNA, (F) *ß-catenin* siRNA, (G) *ß-catenin* siRNA and Wnt3a, or (I) *wnt3* siRNA. (J) Rescue of regeneration after treatment with *wnt3* siRNA and Wnt3a. Arrows: oral siphon pigmented organs. (K) RT-PCR showing *delta1*, *ß-catenin*, and *wtn3* mRNA expression at 6 days post-amputation without corresponding siRNA (-siRNA) and with corresponding siRNA (+siRNA) and *notch* expression without *wnt3* siRNA (−siRNA) and with *wnt3* siRNA (+*wnt3* siRNA). (L) Bar graphs showing the effects of siRNA on percent oral siphon regeneration and rescue by exogenous Wnt3a. The number of animals used in each experiment is shown at the bottom of the bars. Black asterisks indicate significant differences at *P*<0.001 between the respective scrambled controls and the corresponding siRNA treated animals. Red asterisk indicates significant difference at *P*<0.01. Statistics by χ^2^ test and post-hoc Fisher's exact test with Bonferroni correction. Each experiment was replicated at least two times.

To explore the molecular basis of oral siphon regeneration, a pharmacological screen was carried out using small molecule inhibitors of classic signaling systems. In these experiments, animals were pre-incubated with a signaling inhibitor, the oral siphon was amputated, incubation with the inhibitor was continued for 6 days, and the extent of regeneration was examined using the markers described above. SU5402 was used to suppress FGF signaling, cyclopamine and Sant to inhibit Hedgehog signaling, dorsomorphin to inhibit BMP signaling, and FH535 and IWR-1-Endo to interrupt Wnt signaling. Control amputees were treated with DMSO. In addition, the Notch signaling inhibitors DAPT and Compound E, which were previously shown to suppress oral siphon regeneration in *Ciona* (Hamada et al., 2015), were used as positive controls. As shown in Fig. 3A, the FGF, Hedgehog, and BMP inhibitors had no effects on oral siphon regeneration. In contrast, the Wnt and Notch inhibitors were effective in suppressing oral siphon regeneration. These results suggest a role for Wnt signaling in oral siphon regeneration.

RNA interference is effective for knocking down gene expression in adult ascidians (Rosner et al., 2006; Brown et al., 2009; Tiozzo and De Tomaso, 2009; Rinkevich et al., 2010). Therefore, the role of Wnt in distal regeneration was also studied by using short interfering RNA (siRNA) targeting *wnt3* and *ß-catenin* in the *Ciona* Wnt signaling pathway. *Ciona muscle actin 3* (*ma3*) siRNA was used as a negative control. The *ma3* gene encodes a larval type-muscle actin (Kovilur et al., 1993), which is expressed during embryonic development but not in adults (Chiba et al., 2003), and is thus expected to have no effects on regeneration. As a positive control, siRNA corresponding to the Notch pathway gene *delta1*, which is expressed in the regenerating oral siphon (Hamada et al., 2015), was used. Lastly, scrambled sequence siRNAs corresponding to the *delta1*, *wnt3*, and *ß-catenin* genes were used as additional controls. Because *Ciona* constantly filters large volumes of water through the body, an siRNA soaking method was employed in these experiments. Oral siphons were amputated as described above, the amputees were bathed in siRNA, a scrambled sequence siRNA, or DMSO immediately after siphon removal, the original siRNA was exchanged for fresh siRNA at 3 days PA, and regeneration was assayed at 6 days PA using the markers described above. Reverse Transcriptase-Polymerase Chain Reaction (RT-PCR) experiments showed that *delta1*, *ß-catenin*, and *wnt3* expression were reduced by treatment with specific siRNA, although *notch* expression, used as a control, was not markedly affected by *wnt3* siRNA treatment (Fig. 3K). The negative control *ma3* siRNA had no effects on the percent oral siphon regeneration (Fig. 3B,L), whereas the positive control *delta1* siRNA significantly reduced the percentage of amputees with successful oral siphon regeneration (Fig. 3D,L). The *wnt3* and *ß-catenin* siRNAs also strongly suppressed the percentage of amputees showing oral siphon regeneration (Fig. 3F,I,L). None of the scrambled sequence siRNAs had marked effects on the percentage of animals with successful oral siphon regeneration (Fig. 3C,E,H,L). In further experiments, amputees exposed to *wnt3* or *ß catenin* siRNA were co-treated with human recombinant Wnt3a protein and then assayed for oral siphon regeneration at 6 days PA. The results showed that Wnt3a rescued oral siphon regeneration when applied in combination with *wnt3* siRNA (Fig. 3J,L), but not with *ß catenin* siRNA (Fig. 3G,L), indicating that the effects of *wnt3* siRNA on regeneration are reversible by supplying an exogenous Wnt ligand. Wnt3a was not expected to restore the effects of *ß-catenin* siRNA on siphon regeneration because ß-catenin functions downstream in the Wnt pathway. The pharmacological screen and RNA interference experiments support a role for the canonical Wnt pathway in *Ciona* oral siphon regeneration.

### Wnt3a rescues caspase inhibition of distal regeneration and branchial sac homeostasis

The role of Wnt signaling in oral siphon regeneration and homeostasis prompted further studies to determine whether exogenous Wnt can rescue the effects of caspase inhibition, as has been shown for head regeneration in *Hydra* (Chera et al., 2009).

**Fig. 4.**
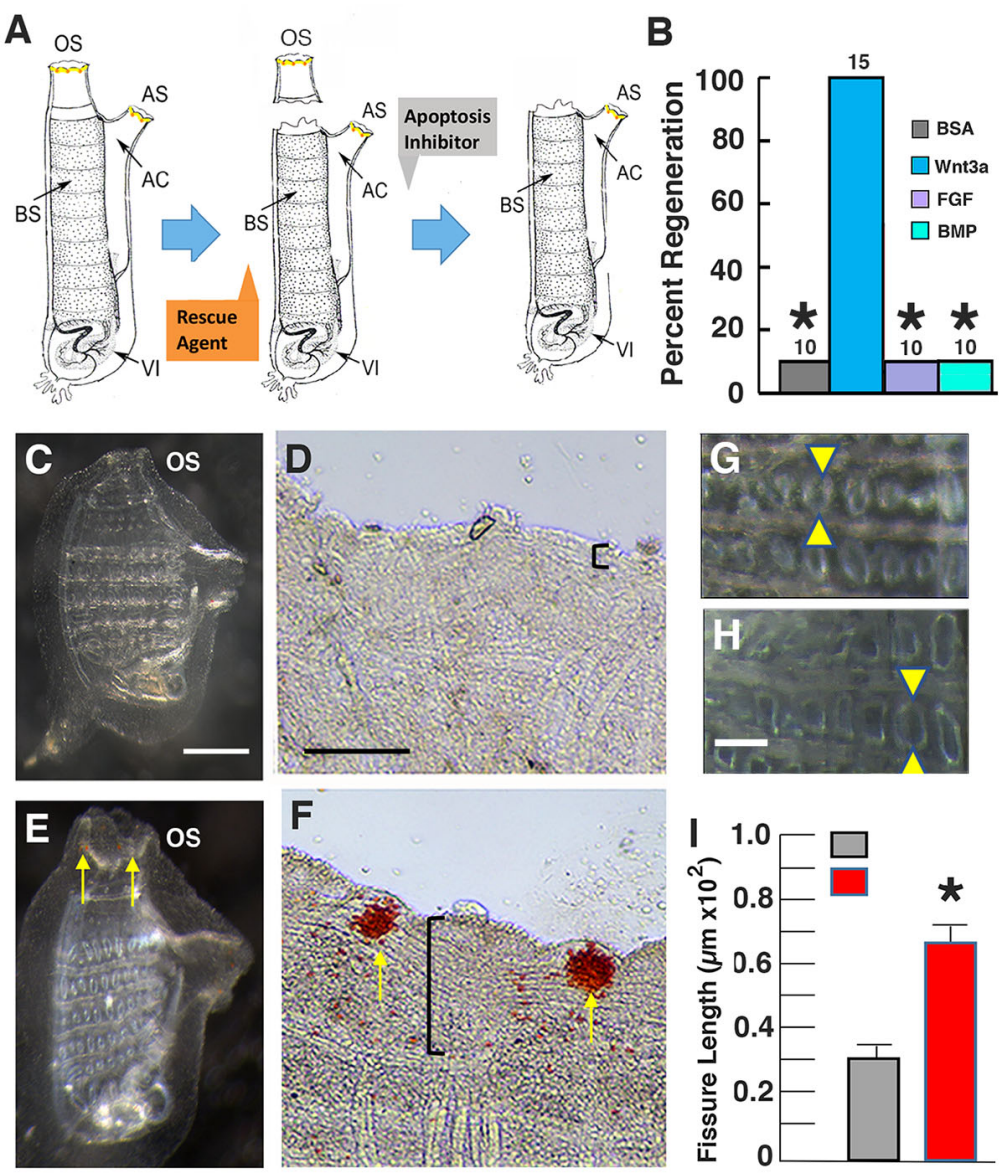
**Wnt rescue of caspase inhibition effects on oral siphon regeneration and branchial sac homeostasis.** (A) Diagram of the rescue experiment. Labels are the same as in Fig. 2J. (B–F) Wnt3a rescue of inhibited oral siphon regeneration. (B) Bar graphs showing the percentage of regenerated oral siphons after exposure to pan-caspase inhibitor Z-VAD-FMK and either bovine serum albumin (BSA, control), Wnt3a, FGF, or BMP for 6-days PA. The numbers of treated animals are shown at the top of each bar. Asterisks indicate significant differences at *P*<0.001 between the control and caspase inhibitor treated animals. Statistics by χ^2^ test and post-hoc Fisher's exact test with Bonferroni correction. (C–F) Oral siphon regeneration in pan-caspase inhibitor Z-VAD-FMK treated animals without (C,D) or with (E,F) Wnt3a. (C,E) Whole animals. (D,F) Magnified areas of siphon margin. Arrows: oral siphon pigmented organs. Vertical brackets: extent of circular muscle band replacement. Scale bar in C: 60 µm; magnification is the same in C and D. Scale bar in D: 10 µm; magnification is the same in D and F. (G–I) Wnt3a rescue of inhibited pharyngeal fissure growth. (G,H) Branchial sacs showing fissure organization in pan-caspase inhibitor Z-VAD-FMK treated animals without (G) or with (H) Wnt3a. Arrowheads: distal (top) and proximal (bottom) ends of the fissures. Scale bar in G: 15 µm; magnification is the same in G and H. (I) Bar graphs showing the mean length of pharyngeal fissures (arrows) in pan-caspase inhibitor Z-VAD-FMK treated animals without or with Wnt3a. Error bars: standard deviation. *N*=12 for both categories. Asterisk: significance at *P*=0.000024. Error bars: s.e.m. Statistics by one-way ANOVA and post-hoc Tukey with Bonferroni correction. Each experiment was replicated at least three times.

To determine the effects of Wnt ligand on apoptosis dependent oral siphon regeneration (Fig. 4A), animals were separated into two groups prior to amputation. One group was bathed in Wnt3a protein for 1 h, and the other group was left untreated. Next, oral siphons were amputated in both groups, pan-caspase inhibitor Z-VAD-FMK was added immediately and was present for 6 days PA, after which regeneration was assayed as described above. Most of the amputees treated with pan-caspase inhibitor Z-VAD-FMK did not regenerate amputated oral siphons (Fig. 4B–D), confirming the earlier results (Fig. 1H,J). In contrast, many of the amputees treated with both Wnt3a and pan-caspase inhibitor Z-VAD-FMK showed oral siphon regeneration, including siphon re-growth, CMB differentiation, and OPO formation (Fig. 4B,E,F). In contrast, similar exposures of amputees to BSA, FGF, or BMP did not rescue oral siphon regeneration in pan-caspase inhibitor Z-VAD-FMK treated amputees (Fig. 4B). The results indicate that Wnt3a rescues the effects of caspase inhibition on oral siphon regeneration.

To determine the effects of exogenous Wnt ligand on apoptosis-dependent branchial sac homeostasis, animals were separated into two groups, and one group was pre-incubated with Wnt3a for 1 h and the other group was left untreated, pan-caspase inhibitor Z-VAD-FMK was added to both groups, incubation was continued for 6 days, and the pharyngeal fissures were measured. Treatment with pan-caspase inhibitor Z-VAD-FMK alone reduced the length of pharyngeal fissures, which was reversed by co-incubation with Wnt3a (Fig. 4G–I), suggesting that exogenous Wnt3a rescued the effects of caspase inhibition on branchial sac homeostasis.

In summary, the results show that exogenous Wnt3a compensates for the negative effects of caspase inhibition on oral siphon regeneration and branchial sac homeostasis, supporting the possibility that apoptosis controls regeneration and branchial sac homeostasis by activating Wnt signaling in *Ciona*.

### Apoptosis is required for progenitor cell survival

**Fig. 5.**
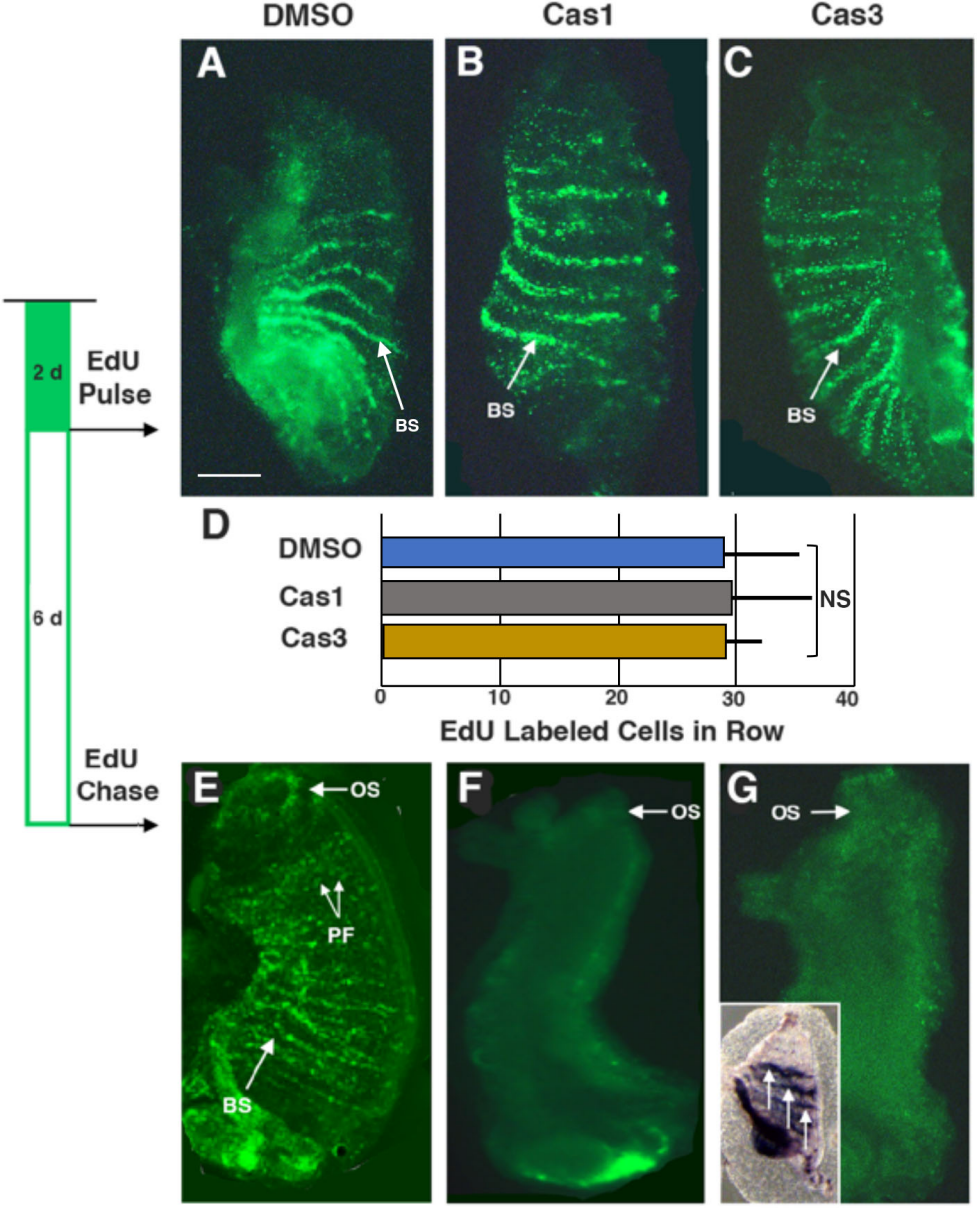
**The relationship between apoptosis-dependent oral siphon regeneration, cell proliferation, and survival in the branchial sac stem cell niche.** Stripes of labeling representing progenitor cells in stem cell niches of the branchial sac after a 2-day EdU pulse followed by a 6-day chase. The EdU pulse-chase schedule is shown on the left. (A,E) DMSO control. (B,F) Caspase 1 inhibitor treatment. (C,G) Caspase 3 inhibitor treatment. (F-inset). Alkaline phosphatase staining of stem cell niches (arrows) in the branchial sac of a caspase 3-inhibitor treated animal subjected to the EdU pulse-chase regime. BS, branchial sac stem cell niche; PF, pharyngeal fissures; OS, oral siphon. (D) Quantification of EdU labeled cells in a vasculature row (see BS) of the branchial sac of DMSO treated controls, caspase 1 inhibitor treated animals, and caspase 3 inhibitor treated animals after the 2-day EdU pulse. *N*=7 for A–C and G. *N*=5 for E and F. Statistics by one-way ANOVA and post-hoc Tukey with Bonferroni correction. NS, no significance. Scale bar in A: 100 µm: magnifications are the same in all large frames.

Adult stem cells are activated to divide and dispatch progenitors for wound repair and siphon regeneration and are also involved in replacement of recycling pharyngeal fissure cells during homeostatic growth in *Ciona* (Jeffery, 2015b, 2019). To determine the role of apoptosis in stem cell activities, oral siphons were amputated, the amputees were immediately treated with caspase 1 or caspase 3 inhibitor and the cell proliferation marker EdU for 2 days PA. Some animals were then fixed and an 5′ ethynyl-2′-deoxyuridine (EdU) pulse was used to determine the effects on progenitor cell proliferation (Fig. 5B,C), whereas other animals were subjected to a 6-day EdU chase to determine the fate of the EdU labeled cells (Fig. 5). DMSO treated controls were also subjected to the EdU pulse-chase labeling schedule (Fig. 5A,E). Quantification of EdU labeled progenitor cells showed no significant differences in the branchial-sac stem cell niches in controls and caspase-inhibitor treated animals during the EdU pulse (Fig. 5A–D), indicating that apoptosis is not required for the activation of stem cell proliferation. However, striking differences were seen between the controls and caspase inhibitor treated amputees following the EdU chase. In controls, EdU labeled cells were chased into the regenerating siphon and cells of the pharyngeal fissures (Fig. 5), as described previously (Jeffery, 2015b, 2019). In contrast, EdU labeling was not visible in the caspase inhibitor-treated animals (Fig. 5F,G), implying that most of the EdU labeled cells perished during the chase. Alkaline phosphatase staining (Jeffery, 2015a) of caspase 3-inhibitor treated animals showed that the stem cells were still present after the EdU chase (Fig. 5G, inset). The results suggest that apoptosis is necessary for progenitor cell survival.

### Apoptosis-dependent Wnt signaling in asymmetric regeneration

**Fig. 6.**
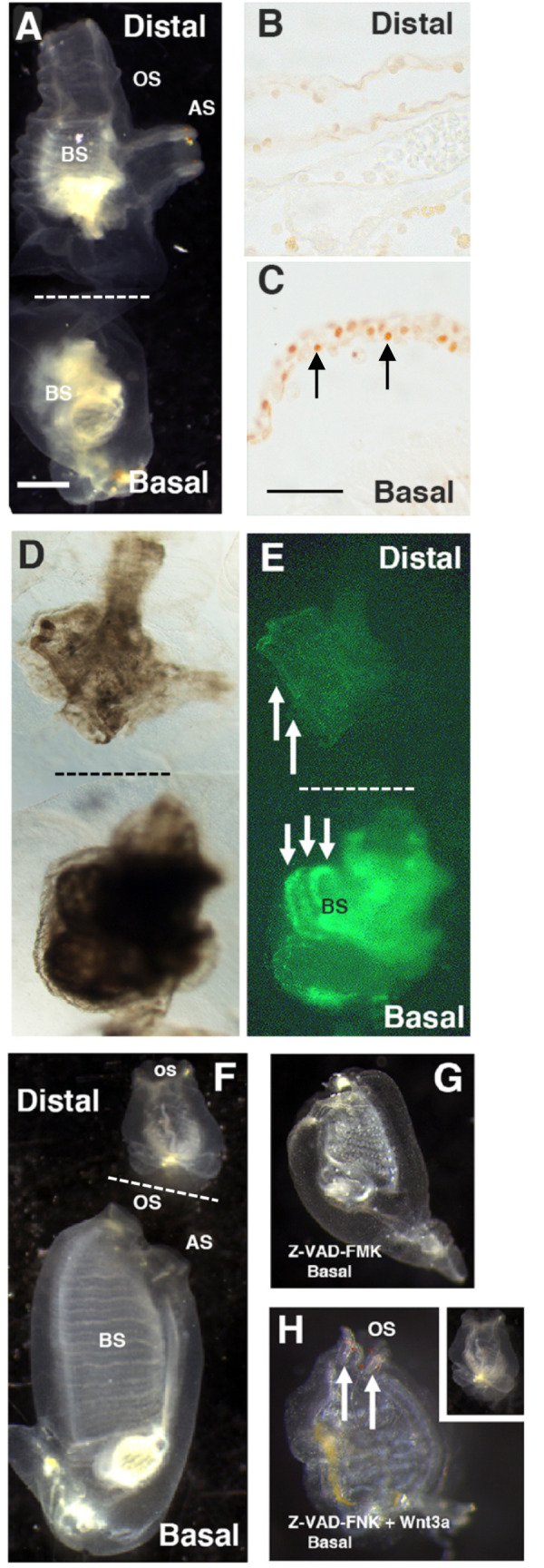
**The roles of apoptosis-dependent regeneration and stem cell activation in mid-body amputation.** (A) Bisected animal immediately after mid-body amputation showing distal and contracted basal fragments. Scale bar in A: 100 µm. Magnifications are the same in A and D–H. (B,C) Sections of the distal (B) and basal (C) fragments 12 h after mid-body amputation showing TUNEL labeled cells (arrows) in the basal but not the distal fragment. Scale bar in C: 10 µm. Magnifications are the same in B and C. (D,E) Bright field (D) and fluorescence (E) images of basal and distal fragments subjected to EdU for 2 days after bisection showing progenitor cell labeling in the branchial sac stem cells of the basal (downward arrows) but not the distal fragment (upward arrows). (F) Bisected control animal after 6 days PA showing the regenerating basal fragment and non-regenerating distal fragment. (G) A basal fragment treated with pan-caspase inhibitor Z-VAD-FMK immediately after mid-body amputation showing the absence of regeneration at 6 days PA. (H) A basal fragment treated with pan-caspase inhibitor Z-VAD-FMK and Wnt3a immediately after mid-body amputation showing rescue of regeneration in the basal fragment (arrows) but not the distal fragment (inset) at 6 days PA. OS, oral siphon; AS, atrial siphon; BS, branchial sac. Dashed lines in A, D–F indicate the bisection plane. Each experiment was replicated at least three times.

In contrast to oral siphon amputation, in which the branchial sac remains unaltered, mid-body amputation produces distal and basal body fragments, each containing about one-half of the branchial sac. However, only the basal fragments regenerate (Hirschler, 1914; Jeffery, 2015b; Fig. 6F). Therefore, further experiments were conducted to determine the role of apoptosis-dependent Wnt signaling in asymmetric regeneration.

Animals were amputated across the mid-body to produce approximately equal-sized distal and basal fragments (Fig. 6A). To address apoptosis, the distal and basal fragments were subjected to TUNEL labeling at 12 h PA. The severed margin of the basal fragments showed a layer of apoptotic cells (Fig. 6C), but no apoptotic cells were detected at the severed margin of the distal fragments (Fig. 6B), showing that apoptosis is restricted to the basal fragments after mid-body amputation. The severed distal and basal fragments were incubated with EdU for 2 days to determine whether branchial sac stem cells were activated to divide in both fragments after mid-body amputation. Progenitor cell labeling was detected in the branchial sac of the basal fragments, but not in the distal fragments (Fig. 6D,E), showing that stem cell activation is unilateral in the basal fragments following mid-body amputation. Mid-body-amputated animals were treated with the pan-caspase inhibitor Z-VAD-FMK to determine whether apoptosis is required for basal fragment regeneration. In DMSO controls, the basal fragments replaced the oral and atrial siphons by 6 days PA, and the distal fragments showed no regeneration at the amputation site (Fig. 6F, Table 1). In contrast, no regeneration occurred in Z-VAD-FMK treated basal fragments at 6 days PA (Fig. 6G, Table 1), or when observed during the next several weeks, indicating that apoptosis is required for basal fragment regeneration. Siphon regeneration also took place in basal fragments treated with both pan-caspase inhibitor Z-VAD-FMK and Wnt3a (Fig. 6H and inset, Table 1), showing that asymmetric Wnt3a rescue occurs after mid-body amputation. However, no growth activity was seen at the amputation sites in distal fragments treated with the caspase inhibitor and Wnt3a, indicating that distal fragments are insensitive to Wnt induced rescue of regeneration.

**Table 1.**
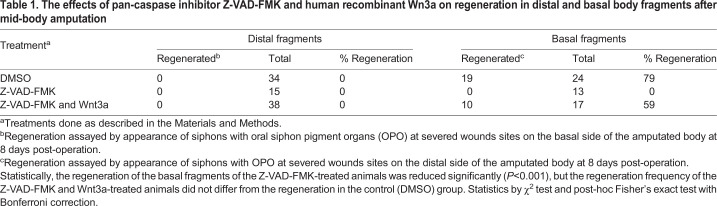
**The effects of pan-caspase inhibitor Z-VAD-FMK and human recombinant Wn3a on regeneration in distal and basal body fragments after mid-body amputation**

The results indicate that mid-body amputation shows the distal fragments of mid-body amputees exhibit no apoptosis at the wound site, no activation of cell proliferation in the branchial sac, and could not be induced to regenerate by exogenous Wnt3a treatment.

## DISCUSSION

**Fig. 7.**
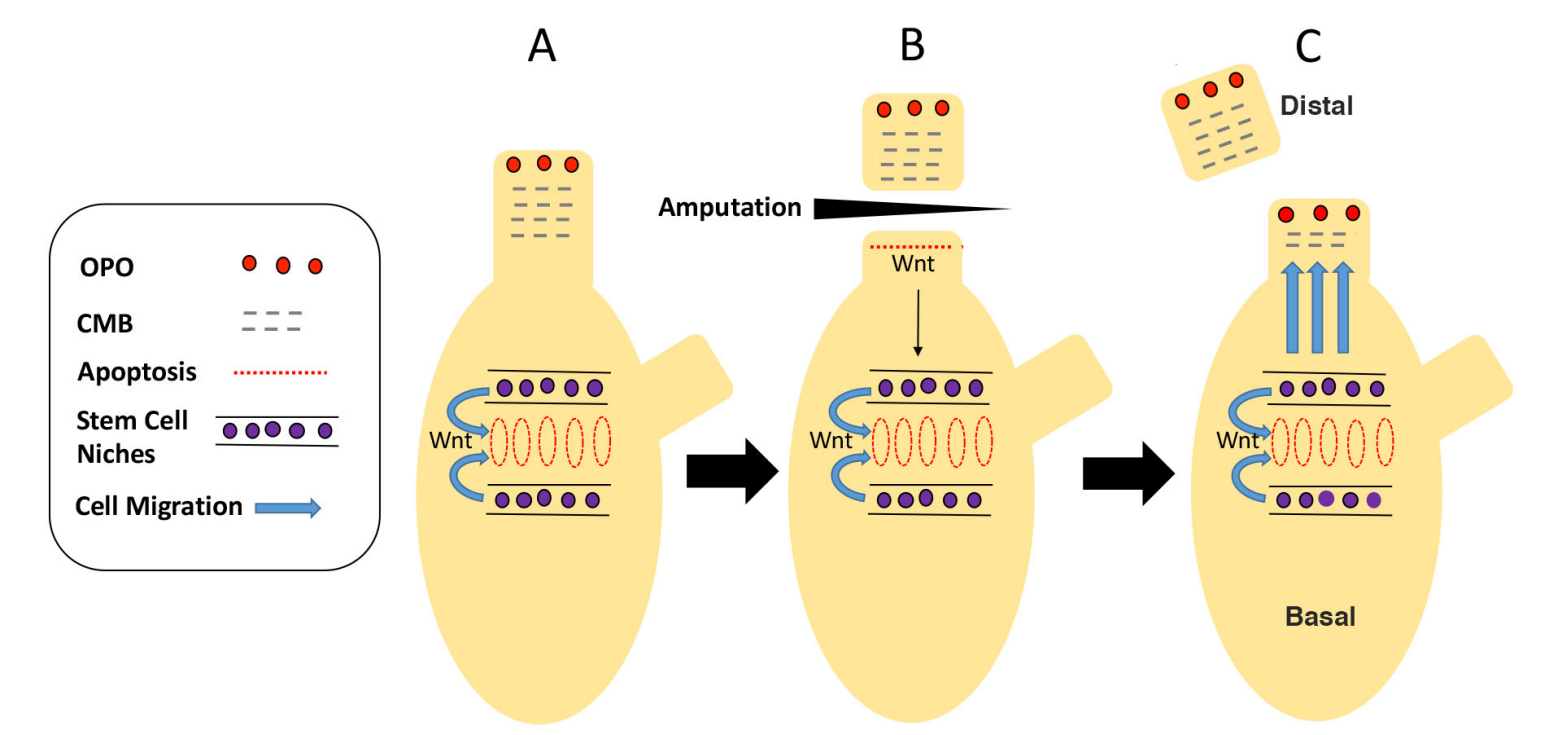
**A model for apoptosis-dependent Wnt signaling in oral siphon regeneration and branchial sac homeostasis in *Ciona*.** (A) Prior to injury, there is no Wnt signaling from the oral siphon stump to the branchial sac stem cell niches. (B) Following oral siphon amputation, apoptosis is activated at the proximal margin of the wound, a Wnt signal is generated as a result of apoptosis, and the signal is relayed basally toward the branchial-sac stem cell niches. (C) During oral siphon regeneration, Wnt signaling maintains the function of branchial sac stem cells, which target progenitor cells to the oral siphon stump for replacement of lost tissues. (A–C) A Wnt signal is continuously generated by apoptotic pharyngeal fissure cells to produce progenitor cells for ciliated cell replacement. OS, oral siphon; AS, atrial siphon; OPO, oral siphon pigment organ; CMB, circular muscle bands.

This study presents new information on the role of apoptotic cell death at the sites of tissue replacement during regeneration and homeostatic growth in the ascidian *Ciona intestinalis*. We previously showed that apoptosis is an early and transient event in the repair and regenerative programs of wounded animals or amputated distal organs, such as the oral and atrial siphons or the neural complex (Jeffery, 2019). As a consequence of such injuries, adult stem cells in the branchial sac are activated to produce progenitor cells, which migrate through the body and replace injured or missing tissues and organs (Jeffery, 2015b). The progenitor cells that replenish blood cells (Ermak, 1976) and rapidly-cycling ciliated cells lining the pharyngeal fissures of the branchial sac (Jeffery, 2019) are also seeded by the continuous proliferation of branchial sac stem cells during homeostatic growth. It is shown here that (1) apoptosis is required for distal regenerative activities and normal homeostatic cell replacement in the branchial sac, (2) Wnt signaling is involved in both of these processes, (3) exogenously applied Wnt ligand can substitute for apoptosis, when the latter is inhibited, and rescue normal regenerative and homeostatic activities, and (4) apoptosis at the wound site is required for the survival, rather than the proliferation, of progenitor cells in the branchial sac stem cell niches. These results suggest an apoptosis-driven Wnt-dependent model for progenitor cell targeting and tissue replacement by adult stem cells during *Ciona* regeneration and homeostasis (Fig. 7).

The caspase system is essential for the initiation and execution of programmed cell death, and its core components are highly conserved during evolution (Cohen, 1997; McIIwain et al., 2013; Bell and Megeney, 2017). Eleven different caspase genes have been identified in *Ciona*, including genes encoding the enzymes responsible for inducing inflammation (e.g. caspase-1) and the initiation and execution of programmed cell death (e.g. caspase-3), suggesting that ascidians utilize inflammation and cell death signaling cores similar to other animals (Terajima et al., 2003). Our approach was to determine the effects of three different specific and general caspase inhibitors on apoptosis associated with *Ciona* regeneration and homeostatic growth. Inhibitors of the inflammatory caspase-1, the apoptotic initiator caspase 3, and the pan-caspase inhibitor Z-VAD-FMK all suppressed apoptotic cell death at the apical margin of the amputated siphon stump and prevented the differentiation of new oral siphon tissues and organs, including the CMB and OPO sensory structures. The pan-caspase inhibitor also blocked the regeneration of basal fragments, which show unilateral apoptosis after mid-body bisection. Apoptosis occurs transiently, beginning shortly after oral siphon amputation and continuing for about 24 h during the regeneration process (Jeffery, 2019 and unpublished). Caspase inhibition was effective in blocking regeneration only when carried out within the first day after amputation, which spans the period of apoptosis, and inhibitor treatment at later times following amputation had no effects on regeneration. These results indicate that apoptotic cell death at amputation sites is required as an early step in the *Ciona* regeneration program. Early apoptosis is also a critical factor in regenerating planarians (Hwang et al., 2004), *Hydra* (Chera et al., 2009), annelids (Fok et al., 2020), newts (Vlaskalin et al., 2004), and *Xenopus* (Tseng et al., 2007). The requirement for apoptosis as an early step of regeneration in *Ciona*, an ascidian chordate, provides additional evidence for evolutionary conservation of the link between apoptosis and regeneration.

Caspase inhibition also prevented apoptosis of cells lining the pharyngeal fissures of the *Ciona* branchial sac. These ciliated cells are responsible for the flow of food-laden seawater through the pharyngeal cavity (Millar, 1953; Manni et al., 2002), turnover rapidly during normal growth (Jeffery, 2019), and like the progenitor cells responsible for regeneration are derived from branchial sac stem cells (Jeffery, 2019). Caspase inhibition reduced pharyngeal fissure growth and prevented water flow and filtration through the body, consistent with a requirement of apoptosis for recycling of ciliated cells produced by adult stem cells. This process is reminiscent of the role of apoptosis in the recycling, replacement, and growth of adult tissues and organs such as the liver in vertebrates (Fogarty and Bergmann, 2017). Other studies have suggested that apoptosis may also be required to promote the migration of primordial germ cell precursors from the larval tail into the head during *Ciona* metamorphosis (Krasovec et al., 2019). Furthermore, during the blastogenic cycle of the colonial ascidian *Botryllus*, massive apoptosis of the regressing zooids of one generation occurs during the migration of germ cells through the blood stream into the next generation of developing zooids (Ballarin et al., 2010). Therefore, it is tempting to postulate a link between apoptosis and cell migration during larval tail retraction, the blastogenic cycle, and regeneration in ascidians.

The results suggest that apoptotic cells may initiate a signaling pathway leading to the activation of adult stem cells in the branchial sac and the replacement or replenishment of lost cells and tissues during regeneration and homeostatic growth. The signal could be produced by the dying apoptotic cells themselves or by nearby living cells. Several lines of evidence indicated that canonical Wnt signaling is involved in apoptosis-driven regeneration and homeostasis in *Ciona*. First, regeneration is blocked by small molecule Wnt inhibitors or by siRNA mediated functional inhibition of the *wnt3* and *ß-catenin* genes. The specificity of the latter results was verified in several different ways, most convincingly by the rescue of *wnt3* siRNA effects on regeneration by exogenous Wnt ligand. Second, exogenous Wnt ligand can rescue the effects of caspase inhibitors on regeneration and branchial sac homeostatic growth. These results are consistent with microarray (Hamada et al., 2015; Gorički, unpublished) and RNA sequencing studies (Spina et al., 2017) showing upregulated expression of Wnt signaling system components during *Ciona* oral siphon regeneration.

Apoptosis-driven regeneration based on Wnt signaling is also involved in *Hydra* regeneration (Chera et al., 2009), and Wnt signaling has been shown to be central to regeneration throughout the animal kingdom (Whyte et al., 2012). Therefore, our results align *Ciona* regeneration with conclusions obtained from experiments on other regenerating systems. In these situations, it has been shown that apoptosis mediates regeneration by activating the proliferation of stem cells, which has been referred to as a caspase- or an apoptosis-driven proliferation process (Fogarty and Bergmann, 2017). However, according to our results, the situation in *Ciona* may be different. We found that caspase inhibition did not affect the proliferative activity of branchial sac stem cells, but instead appeared to affect the survival of progenitor cells derived from the stem cell niches. At least in *Ciona*, the effects of caspase inhibitors on the stem cell niches are thus more accurately described as caspase- or apoptosis-driven cell survival. We do not currently understand how the progenitor cells disappear during the course of the chase experiments, although it is conceivable that they die through apoptosis and are rapidly cleared from the body by circulating phagocytes.

Intrinsic differences between oral siphon and mid-body amputation, most importantly the presence of part of the branchial sac and its stem cell niches in distal fragments after the latter operation, lead to interesting conclusions about the asymmetric regeneration process at wound sites in *Ciona*. First, apoptosis is unilateral, occurring at the severed margin of the basal but not the distal fragments. Therefore, there seems to be no possibility of apoptosis-driven downstream events in the distal fragments, and this may be a crucial factor in their inability to regenerate. The complete explanation is probably more complex, however, because application of the Wnt ligand, which rescued caspase-blocked regenerative activity in the oral siphon stumps and in the basal fragments produced by mid-body amputation, did not have the same effects on the distal fragments after mid-body regeneration. Second, although the distal fragments of mid-body amputations contain a large part of the original branchial sac, EdU labeling showed that stem cells in this part of the branchial sac were not activated to divide and produce progenitor cells, as occurred in the part of the branchial sac that remains in the basal fragments, and this may be another reason why distal fragments are unable to regenerate. This result also shows that the presence of a branchial sac is not sufficient in itself for regeneration. The first and second conclusions above suggest that additional signaling, possibly arising from somewhere in the basal portion of the animal, may also be involved in apoptosis, stem cell proliferation, and regeneration. Future studies with the non-regenerating distal fragments, in particular the induction of ectopic apoptosis, may provide further insights into the mechanisms of *Ciona* regeneration.

## MATERIALS AND METHODS

### Animals

*Ciona intestinalis* was collected at Sandwich Harbor near Woods Hole, MA, USA, or raised from fertilized eggs at Station Biologique, Roscoff, France. The larvae were allowed to attach and undergo metamorphosis on plastic Petri dishes. The Petri dishes with attached juveniles were placed on racks in aquaria, fed daily with green algae, and small adults were raised to the desired size for operations (1–2 months old with about 8–16 transverse vessels in their branchial sacs) in aquaria with running sea water. For the pharmacological screen, 6–8 cm adults were used, which were farmed in the laboratory from fertilized eggs or freshly collected from the wild.

### Operations

Animals were anesthetized by treatment for 15–20 min with 0.2 mg/ml tricaine methane-sulfonate (MS222; Sigma-Aldrich, St. Louis, MO, USA) buffered in Millipore filtered sea water (MFSW). Operations were carried out using straight-bladed micro-cautery scissors or fine dissection scissors (Fine Science Tools, Foster City, CA, USA). Oral siphons were amputated by severing perpendicular to the long axis at a position immediately below the ring of tentacles, as described previously (Auger et al., 2010). Mid-body amputations were made through a plane perpendicular to the longitudinal axis at the level of the rectal opening into the atrial cavity. Operated animals were cultured while attached to Petri dishes, which were placed on racks in running sea water aquaria, or were detached and raised for 6–8 days post-amputation (PA) in plastic cell wells containing MFSW.

### Caspase inhibition

Animals were treated with 30 µM caspase-1 inhibitor YVAD-CHO (Calbiochem, San Diego, CA, USA), 30 µM caspase-3 inhibitor DEVD-CHO (Calbiochem), or 1.5 µM pan-caspase inhibitor Z-VAD-FMK (R&D Systems, Minneapolis, MN, USA). Stock solutions were prepared in dimethyl sulfoxide (DMSO; Sigma-Aldrich), frozen, and thawed before each experiment. The effective dose of the caspase inhibitors was determined empirically from the effects of a dilution series on survival and oral siphon regeneration. Caspase inhibitor incubations were done at 16–18°C in the dark.

### Apoptosis detection

Apoptotic cells were detected by terminal deoxynucleotidyl transferase dUTP nick end labeling (TUNEL) as described previously (Jeffery, 2002). To detect apoptosis after oral siphon amputation, amputees were anesthetized at 12 h PA as described above, their tunics were removed by dissection, the denuded animals were fixed in 4% paraformaldehyde (PFA) for 14 h at 4°C, and washed three times in phosphate buffered saline (PBS), permeabilized in 0.5% Triton X-100, and then washed three more times with PBS. The samples were processed for detection of apoptotic cells with Alexa Fluor azide 488 using the Click-it Plus TUNEL kit (Thermo Fisher Scientific, Waltham, MA, USA) according to the manufacturer's instructions, and imaged by fluorescence microscopy. Apoptosis was quantified in flat mount preparations (Auger et al., 2010) by manually counting TUNEL labeling in 200 µm^2^ areas centered along the edge of the siphon stump. To detect apoptosis in the pharyngeal fissures of the branchial sac or in the severed ends of distal and basal fragments after mid-body amputation, TUNEL was conducted using the In Situ Cell Death Kit (Roche Applied Science, Indianopolis, IN, USA) as described previously (Jeffery, 2002, 2019) using animals processed as above. The specimens were post-fixed in 4% PFA in PBS overnight at 4°C, embedded in Paraplast, sectioned at 10 µm, the sections were attached to gelatin subbed glass slides, and unstained sections were imaged by microscopy.

### Carmine particle assay for branchial sac filtration

Carmine powder (Thermo Fisher Scientific, Waltham, MA, USA) was pulverized with a mortar and pestle. Animals treated with DMSO or caspase inhibitors were mixed with a suspension of 0.1 mg/ml carmine particles in MFSW and incubated for 5 h at room temperature. At the end of the assay, animals were photographed using dark field optics.

### Pharyngeal fissure analysis

DMSO and caspase-treated animals were anesthetized as described above and flattened to a thin layer on glass microscope slides by withdrawing most of the MFSW. The pharyngeal fissures were measured along their longitudinal axis using an optic micrometer. Ten fissures were measured along the mid-body row and the values averaged for each animal.

### EdU pulse-chase labeling and quantification

Animals treated with DMSO or caspase inhibitors were incubated with 200 µmol/l 5′ ethynyl-2′-deoxyuridine (EdU; Invitrogen, Carlsbad, CA, USA) for 2 days PA at 18°C in MFSW for the EdU pulse. The EdU was chased by five successive washes in MFSW and subsequent culture in MFSW without EdU for 6 days. EdU pulse and chase labeled animals were relaxed by treatment with 2–4 crystals of menthol (Sigma-Aldrich) for 30 min at room temperature, fixed in 4% PFA for 14 h, washed three times in PBS, permeabilized in 0.5% Triton X-100 at room temperature, washed three more times with PBS, processed for EdU detection with Alexa Fluor azide 488 using the Click-it imagining kit (Thermo Fisher Scientific), as described previously (Jeffery, 2015b), and imaged by fluorescence microscopy. EdU labeled cells were quantified after the 2 h pulse by manual counting along the fifth horizontal row of vasculature from the basal end of the branchial sac on one side of the plane of bilateral symmetry (see BS in Fig. 5A–C).

### Alkaline phosphatase staining

Animals from the EdU pulse-chase experiments were relaxed in menthol (see above) fixed in 4% formaldehyde for 1 h at room temperature, washed three times in PBS, and then treated with BCIP-NRH (Invitrogen) for 15–30 min at room temperature in the dark (Jeffery, 2015b). After development of purple color, the animals were washed three times in PBS and imaged.

### Pharmacological screen

The pharmacological screen was carried out using adult animals 6–8 cm in length. The FGF signaling pathway inhibitor was SU5402 (Tocris Bioscience, Bristol, UK) (5 μM) (Grand et al., 2004), the Hedgehog signaling pathway inhibitors were cyclopamine (Tocris Bioscience) (20 μM) (Chen et al., 2002a) and Sant-1 (Tocris Bioscience) (15 µM) (Chen et al., 2002b), the BMP signaling pathway inhibitor was dorsomorphin (Tocris Bioscience) (5 µM) (Morizane et al., 2011), the Notch signaling pathway inhibitors were DAPT (Tocris Bioscience) (12 μM) (Dovey et al., 2001) and Compound E (Abcam, Cambridge, MA, USA) (1 μM) (Seiffert et al., 2000), and the Wnt pathway signaling inhibitors were FH535 (Santa Cruz Biotechnology Inc., Dallas, TX, USA) (1.2 μM) (Handeli and Simon, 2008) and IWR-1-Endo (Santa Cruz Biotechnology) (2.5 μM) (Chen et al., 2009). Stock solutions were prepared in DMSO. The effective concentrations were determined by assaying survival and oral siphon regeneration capacity in serial dilutions. Animals were pre-incubated with an inhibitor for 12 h at 16–18°C prior to oral siphon amputation. After oral siphon amputation, the amputees were treated with an inhibitor for 8 days at 16–18°C with fresh changes of inhibitor added every 2 days. The unoperated controls were treated with DMSO in the same final concentration as in the inhibitors.

### Short interfering RNA treatment

Treatment was carried out using siRNAs designed by Invitrogen using NCBI sequence information for *Ciona intestinalis*. The sequence of *ma3* siRNA was 5′-CUUGUUUGAUGAUGUACCUAAGAC-3′, the sequence of *delta1* siRNA was 5′-CCAGUGAAGGCUCUUUCCAAUUGA-3′, the sequence of the scrambled control *delta1* siRNA was 5′-CCAAAGCGGUCUCUUAACUUGUGAA-3′, the sequence of the *ß-catenin* siRNA was 5′-CCAAGUGGUUGUUCAACAATT-3′, the sequence of the scrambled control *ß-catenin* siRNA was 5′-CCAGUUGUUGGUUUACAAGCAATT-3′, the sequence of *wnt3* siRNA was 5′-GCAGUAACGUCCUGGCUAATT-3′, and the sequence of the scrambled control *wnt3* siRNA was 5′-GCAGCAAUCCUCGGUGUAATT-3′. The siRNAs were diluted in RNase-free water to prepare 100 µM stock solutions, which were stored at −20°C prior to use. The effective siRNA concentrations were determined empirically by assaying the effects of a dilution series on survival and oral siphon regeneration. Animals were treated with 2 µM siRNA in MFSW immediately after amputation. At 3 days PA, the animals were rinsed once with MFSW and incubated in fresh 2 µM siRNA, and regeneration was assayed after 6 days of culture at 18°C.

### Reverse transcriptase polymerase chain reaction

Total RNA was isolated from 6-day regenerating animals incubated with or without siRNA using the RNeasy micro kit (Qiagen, Valencia, CA, USA) following the manufacturer's protocols. cDNA was synthesized using the SuperScriptTM III First-Strand Synthesis Super Mix Kit and oligo (dT)_20_ primers (Life Technologies, Carlsbad, CA, USA). RC-PCR was performed using the PCR Master Kit (Roche) under the following cycling conditions: one cycle for 2 min at 94°C, five cycles for 1 min at 94°C, 2 min at 35°C and 3 min at 72°C, five cycles for 1 min at 94°C, 2 min at 45°C and 3 min at 72°C, 20 cycles for 1 min at 94°C, 2 min at 55°C and 3 min at 72°C, and one cycle for 10 min at 72°C. The primers used for *delta1* amplification were 5′-GAAACGGTGTAACTGGAGCC-3′ (forward) and 5′-GGTCTCTCACAAGTTCCATGC-3′ (reverse), for *ß-catenin* amplification were 5′-TGGCACAAAATGCTGTTCGG-3′ (forward) and 5′-CTCGTTCTCGATGAGGTCGG-3′ (reverse), for *wnt3* amplification were 5′-CGATAAAATGGCCAGCCTGC-3′ (forward) and 5′-CAAACTCAGAACTCCGGCCT-3′ (reverse), and for *notch* amplification were 5′-CGGGCAAGAAACCTCGTCGTCA-3′ (forward) and 5′-CTGAACTGGTGCGGAACCCCT-3′ (reverse).

### Wnt3a rescue

Rescue experiments were carried out at 16–18°C in the same way as in the siRNA and caspase inhibitor experiments. Animals were pre-incubated with 150 ng/ml Wnt3a (R&D Systems) in MFSW for 1 h prior to amputation, amputated, then incubated with fresh 150 ng/ml Wnt3a along with an siRNA or a caspase inhibitor at 18°C as described above. As controls, amputated animals were treated with 150 ng/ml Bovine Serum Albumin (BSA; Thermo Fisher Scientific), human recombinant BMP-2 (Advent Bio, Elk Grove Village, IL, USA) or human recombinant FGF-10 (Advent Bio) using the same regime as described for Wnt3a.

### Statistics

Statistical analysis was carried out by one-way ANOVA and post-hoc Tukey with Bonferroni correction as described in the figure legends after application of the Shapiro-Wilk test to determine the normality of data distribution. Regeneration frequencies under different conditions were evaluated by the χ^2^ test and post-hoc Fisher's exact test with Bonferroni correction (Shan and Gerstenberger, 2017) as described in the figure legends.
